# Editorial

**Published:** 1844-08-10

**Authors:** 


THE MEDICAL EXAMINER.

PHILADELPHIA, AUG. 10, 1844.

Several notices of late publications, and other matters crowded out of the present number, shall appear in our next.

---

We are indebted to Dr. Mendenhall, of Cincinnati, for No. 1, New Series, of the *Journal of Health*, a neat periodical published in that city. It is "conducted by the Physicians of the Cincinnati Dispensary and Vaccine Institution." The editors for July, August, and September, are Drs. Vattier, Stuart, and Warder. The plan of the Journal is similar to that of the Journal of Health published in this city some years since, under the able editorial management of Drs. Bell and Condie. The gentlemen concerned in the present publication are altogether competent to the task they have assumed, as the number before us abundantly proves. The original matter is good, and the selected articles are various and apposite.

---

### ALBANY COUNTY MEDICAL SOCIETY.

In a former number, (June 15th,) when noticing the annual report of the transactions of the New York State Medical Society, we took occasion to express our disbelief in the efficacy of all legislative enactments for the suppression of Quackery in a country like ours, in which public opinion is more omnipotent than law, and at the same time we intimated our full conviction that the only mode of distinguishing, or rather enabling the community to distinguish, between the physician and the ignorant pretender, consists in the thorough education and honorable bearing of those who aspire to be regarded as regular members of the profession. That, and that alone, can preserve the profession from sinking to a level with the fools and knaves who, under every imaginable pretext, seek to tamper with the lives and limbs of the people.

The legislature of New York, on the 6th of May last, passed a law relieving all unlicensed practitioners from the penalties prescribed in former acts, placing the practice of medicine in that respect on a level with ordinary trades and occupations. Any person may practice medicine now in the State of New York that pleases, and charge and recover for his services as in ordinary contracts; being liable, however, to civil and criminal prosecutions for mal-practice, gross ignorance, and immoral conduct.

The Albany County Medical Society, it seems, appointed a committee consisting of Drs. Hun, Wing, and Cogswell, to consider and report upon this law and its effects upon their corporate rights and duties, and as to whether any action was necessary in relation thereto. This committee made a very able report, at a special meeting of the society held on the 25th of June, from which we make the following extracts:

"Previous to the passage of this act the law prescribed the mode of becoming a licensed practitioner of Medicine, and conferred on such, and on the Botanic doctors the exclusive right to practice. Since the passage of this act, the law still prescribes the mode of becoming a licensed practitioner, but gives to all persons of whatever age or sex or education, the right to practice, and to enforce the payment of compensation for their services.

Hence, although the organization of the County and State Societies is left as before, it is no longer obligatory on those who practice Physic and Surgery to become members of the County Societies, nor to go through the course of study and the examination requisite for admission into those Societies. They have become voluntary associations, which give to their members the title of licensed practitioners, but confer on them no legal rights.

Such is the operation of this act on the laws regulating Medical Practice. * * * *

It might be supposed, that when men have the choice before them of educated physicians, presenting evidences of their qualifications, and of others whose main titles seem to be their ignorance and impudence, they would not hesitate to have recourse to the former. But sad experience shows that this is far from being true. We find that men who conduct all their other affairs with prudence and discretion, are willing to abandon a medical attendant of tried skill and character, for any juggling mountebank whose pretensions would only excite a smile, were it not for the deplorable results to which they give rise.

Struck with this sad spectacle of human credulity and folly in cases in which such important interests are involved, legislators have thought that it was not sufficient to provide educated physicians, and to give the public the means of recognizing them, but have passed laws prohibiting all but regular physicians from practising. These laws are founded on the assumption, that it would be so absurd to have recourse for medical aid to an ignorant person, when it is possible to procure the services of an educated physician, that those who might be tempted to do so must be treated as incompetent to manage their own concerns. To prevent them, therefore, from indulging in such folly, all irregular practice is prohibited under certain penalties.

The laws for educating and organizing a body of physicians were intended to give men the means of acting prudently; the prohibitory laws were intended to compel men to act prudently.

So long as public sentiment accords with this view of the legislator, the operation of these prohibitory laws is salutary; while only a very few silly persons prefer to have recourse to men out of the profession for relief, it seems proper to protect them against their own bad judgment, just as minors and imbecile persons are not allowed to make contracts by which they might be swindled by knaves. But unfortunately a large portion of the public think that education and science are not necessary to qualify men for medical practice. Numerous sects have sprung up, pretending to cure diseases by various processes, more or less ridiculous, but all agreeing in this one point, that it is not necessary to pass thro' the regular course of studies required by law, but that there is a royal road to medical practice which renders such drudgery useless. These sects, absurd as their doctrines may be, have succeeded in gaining followers among the public, and the effect of these restrictive laws, if enforced, must be to prevent all their followers from procuring the kind of medical aid which they prefer. Besides, it must be remarked that those who are thus placed under Legislative tutelage, are not exclusively the ignorant or imbecile, but that they number in their ranks many persons of education and sagacity who manage all their other affairs with sufficient acuteness and discernment.

However absurd the opinions and conduct of these men may appear to us, we have not, for that reason, the right to impose on them our ideas of wisdom. If, for example, a full grown man, who is capable of managing his own business, chooses to call in, to reduce a dislocation, a natural bone-setter who avows that he has never seen a skeleton, in preference to a surgeon who has devoted himself to the study of such accidents, we may deplore his folly, and endeavor to persuade him to act more prudently, but we ought not to use compulsion either directly or indirectly. If his conduct is foolish, he alone suffers from it, and as we are not responsible for his folly, we have no right to prevent him from indulging in it.

On this point we have the misfortune to differ with some for whose opinions we have great respect, and we wish to be well understood. None can be more deeply impressed than we are with the immense amount of mischief inflicted on community by irregular practitioners of medicine. We feel indignant at the base deception they daily practise under our eyes, and we pity their dupes. We all alike agree in deploring the evil, but there is some difference of opinion as to the remedy. The experience of the past satisfies us that legislative wisdom never can restrain individual folly; that all that legislation can do in such matters is to give to all the means of knowing the character of those to whom they may apply, and thus enable them to act with a full knowledge of the circumstances, and leave the rest to each man's own wisdom and prudence.

We are accustomed to apply this principle to other cases of a like nature. Absurd and mischievous religious systems sometimes spring up. We are pained to see men led away by vile superstitions or fall victims to the arts of designing leaders, yet we do not attempt to put down such systems by law, because we do not think it right to impose our religious views upon others, and because we know that any such attempt would only serve to confirm them in error. So too in matters of ordinary business, the law protects men against imposition so far that if one, in making a bargain, is deceived by false representations, the law would give him redress, but if, with a full knowledge of the facts, one enters into a foolish bargain, he must abide by the consequences. There is no reason why the same principle should not be applied to the medical practice.

But even admitting that these restrictive laws are founded on principles of sound policy and justice, there is still one objection which is unanswerable. It is entirely impossible in this country to enforce them. For many years they have been in existence, and yet men have practised under our eyes openly and avowedly in violation of them, and in no one instance has the penalty been enforced. As to the disability of recovering payment for their services by legal process, it has had quite as little influence, for we think it is altogether probable that botanic doctors and homeopathists and other quacks have been quite as well paid as the regular practitioners.

The practical operation of these laws was rather favourable to the class of irregular practitioners. The penalty they imposed was never regarded, the disability of collecting debts afforded a pretext for demanding payment in advance, and gave to their demands the character of debts of honour. Besides this, they put it in the power of the quacks to raise a cry of persecution, and represent the regular profession as greedy monopolists, and thus excite some feeling in their favour among weak and credulous people. A clamor for the repeal of these laws was kept up for the purpose of advertising the system, rather than of obtaining any rights about which they really cared, and since the repeal has been obtained they will have to devise some new plan to wriggle themselves into notice.

It will be remarked, that in all our reasoning on the subject of these restrictive laws, we have considered them as designed for the good of the public and not of the profession. This is undoubtedly the only ground on which they can be defended. The object of those who enacted them, was to protect the public against the ignorance and rapacity of quacks, and not to protect the profession in a monopoly of practice to be enjoyed for the benefit of its members. If in the repeal of these laws a wrong was committed, the public and not the profession must be considered the injured party.

It behooves us neither to claim as a right nor to ask as a favour any exclusive privilege, which is opposed to or which is not directly conducive to the public good. If these restrictive laws are not called for from considerations of public safety, then there should be no opposition on our part to that repeal. It is certain, that no class of community are so little liable to be injured by quacks as physicians who know how to avoid them.

This point has been lost sight of in the discussions on the subject in the legislature and elsewhere, and we are anxious to bring it clearly in view, because it does not comport with the dignity of our profession to appear to be engaged in a selfish contest for privilege with the different bodies of quacks which infest the community. * * * * *

To resume. We consider that the great end of legislation in medical practice should be to provide a body of competent physicians and to give the public the means of recognizing them, leaving to the prudence of individuals to choose discreetly; and that all attempts to coerce people to discretion are wrong in principle and unsuccessful in practice.

We are now prepared to examine the question, whether under the circumstances any action of the Society is called for?

We have expressed our views as regards the restrictive laws. Whatever difference of opinion may exist as regards the general policy of such laws, there is one point on which all must agree. It is utterly impossible to enforce them so long as they are not in accordance with public sentiment. We would therefore be exceedingly sorry to see the profession again entering into a contest with Thomsonians and other persons of that class, for the sake of restoring a law which we know beforehand cannot be executed, and which serves as a pretext for quacks of all kinds to raise a cry of persecution, and to represent the profession as made up of selfish monopolists—a contest in which defeat would be mortifying and success would bring no real advantage. * * *

Now that all restrictions on practice are removed, it will be practicable to raise the standard of admission into the County Societies without exciting any well founded opposition. These Societies are now voluntary associations, into which those who find the requirements too high need not enter. A well matured plan, which would increase the amount of requisitions without putting it at a point unattainable at the present time, would no doubt be favorably received by the profession.

We would then say, in conclusion, we have laws enough and good laws. Quackery must be suppressed, not by legislation, but by enlightening the public as to its dangers. The dignity and respectability of our profession is to be promoted, not by asking for legal privileges, but by an increase of individual zeal and a more cordial co-operation. It is a great error to suppose that the repeal of the restrictive laws puts the physician on a level with the quack and takes away the barrier which separated them. The barrier which effectually separates the two classes is formed by the higher attainments and honorable deportment of the members of the former, and this is the barrier which it depends on us to make higher and stronger. It is one which quackery will not surmount, and which no legislative enactment can break down."

We have thus given the most material parts of this able report, and only regret that we have not space for the whole: it is a true and manly exposition of the real position of the profession on this subject, expressed with a clearness and force that must carry conviction to the minds of all who read it.

